# *Ayenia nathaliae* (Malvaceae, Byttnerioideae), a new species from Colombia and three new combinations

**DOI:** 10.3897/phytokeys.273.188574

**Published:** 2026-04-15

**Authors:** Laurence J. Dorr

**Affiliations:** 1 Department of Botany, MRC-166, National Museum of Natural History, Smithsonian Institution, P.O. Box 37012, Washington, D.C. 20013–7012, USA National Museum of Natural History, Smithsonian Institution Washington United States of America

**Keywords:** *
Ayenia
*, *
Byttneria
*, Byttnerioideae, Colombia, Malvaceae

## Abstract

*Ayenia nathaliae* Dorr, **sp. nov**. is described and illustrated, and its relationships are discussed. It is known only from the Magdalena Medio subregion of Antioquia department, Colombia. Morphologically, it is similar to *A. saligna* Dorr, another Colombian endemic from the Bajo Cauca and Nordeste subregions of Antioquia and also restricted to riparian habitats. The new species is tentatively assigned to *A.* subsect. *Stipulares* Cristóbal, an early diverging lineage of species within *Ayenia* s.str. found in northern South America and Central America. However, the boundaries of this subsection and other subsections are unclear. In addition, after reviewing the species of *Ayenia* L. and *Byttneria* Loefl. known from Colombia, three additional species of the latter genus are transferred to the former.

## Introduction

*Ayenia* L. (Malvaceae, Byttnerioideae) broadly construed is a genus of ca. 217 species (POWO 2026) found in the U.S.A., Mexico, Central America, the West Indies, South America, Africa, Madagascar, India, southeast Asia, and Malesia. When last revised by Cristóbal (1960), *Ayenia* s.str. included 68 species and was restricted to the Americas. Although Cristóbal (1960, 1976) maintained *Ayenia* and *Byttneria* Loefl. as distinct albeit closely related genera, Sandwith (1939), while describing *A. praeclara* Sandwith thought that *A. praeclara* and *A. stipularis* Triana & Planch., both of which are arborescent and possess foliar nectaries, were “bridge-species” between *Ayenia* and *Byttneria*, genera that he thought might just as well be united. After Whitlock and Hale (2011) published molecular data that showed *Ayenia* embedded in the genus *Byttneria*, Christenhusz et al. (2018) effected the transfer of most species of the latter genus to the former. This expanded concept of *Ayenia* was accepted in the most recent conspectus of the Malvaceae (Colli-Silva et al. 2025).

The following new species was discovered while examining undetermined Malvaceae at the New York Botanical Garden (NY). Initial impressions were that the specimen might be *Ayenia saligna* Dorr, also described from Colombia, but close examination confirmed that it is a distinct species.

## Taxonomic summary

### Ayenia nathaliae

Taxon classification

Plantae

MalvalesMalvaceae

Dorr sp. nov.

D1ED204F-1288-5D4B-8D1E-63024793ACB6

urn:lsid:ipni.org:names:77378628-1

Fig. 1

#### Diagnosis.

*Ayenia nathaliae* Dorr differs from *A. saligna* Dorr in having smaller leaf blades (0.9–3.3 × 0.2–0.4 versus 2.5–7.5 × 0.5–1 cm) that are narrowly oblong (versus lanceolate) with abruptly apiculate (versus long acuminate) apices and a mostly entire (versus typically denticulate) margin, and in lacking (versus possessing) an abaxial appendage on the petal lamina and in lacking (versus possessing) cilia on the basal margin of the petal lamina lobes.

**Figure 1.**
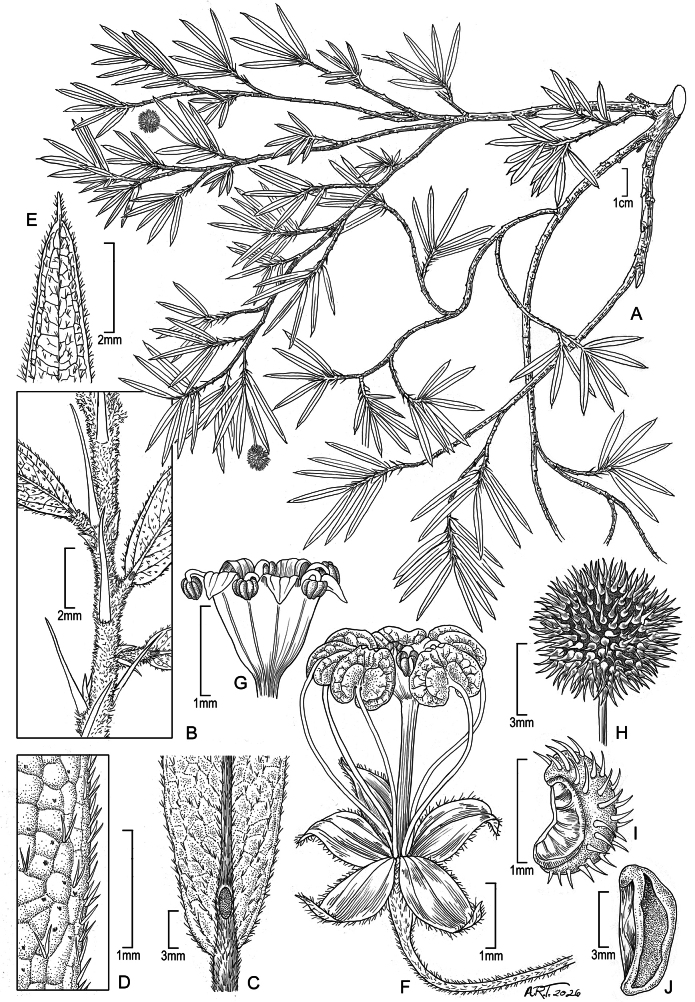
*Ayenia nathaliae* Dorr, sp. nov. **A**. Habit; **B**. Detail of branch showing acicular stipules; **C**. Abaxial view of leaf base showing the solitary foliar nectary on the midvein; **D**. Adaxial view of entire leaf margin showing pubescence; **E**. Adaxial view of leaf apex showing long apiculus; **F**. Complete flower showing the 5-parted calyx, 5 petals (filiform claws and broadly ovate laminas with rounded basal lobes), and androgynophore; **G**. Campanulate staminal column showing 3-thecate anthers alternating with triangular staminodia; **H**. Mature capsule; **I**. One carpel after dehiscence; **J**. Seed (Voucher: *R. Callejas et al. 9347* (NY)).

#### Type.

Colombia. • Antioquia: Municipio Puerto Berrío, Vereda Bodegas, sitio San Juan de Bedout, Quebrada de San Juan, 300 m alt., 6°37'N, 74°30'W, 4 Mar 1990 (fl, fr), *R. Callejas, F.J. Roldán, V. Maza & J.G. Estrada 9347* (holotype: HUA [72577]; isotypes: MO [MO-2384406], NY [01400051]!).

#### Description.

Shrubs, 1 m tall; profusely branched. Young branchlets densely pubescent with simple and bifurcate hairs; older stems glabrate, black when dry. Leaves alternate, internodes short, 1–2 mm long; leaf blades narrowly oblong, 0.9–3.3 × 0.2–0.4 cm, bases cuneate, margin entire, occasionally toothed toward the apex, apices abruptly apiculate, blades scabrous throughout, with simple and bifurcate hairs above and below largely confined to the midveins and margins but also found sparingly on secondary veins below, minute black glandular dots scattered throughout above and below; leaf midveins raised above and below, abaxial midvein with an inconspicuous multi-aperturate nectary ca. 1 (or less) mm from the leaf base, nectaries elliptic, ca. 1 mm long; secondary and tertiary veins also visible below with 10× magnification; petioles ca. 1 mm long, densely pubescent; stipules acicular, often slightly curved, ca. 3 mm long, persistent. Flowers solitary, axillary; peduncles to ca. 9 mm long, articulated ca. 4 mm below the calyx, pubescent with short bristle-like simple or fasciculate hairs, subtended by 3, acicular bracts, sparingly pubescent, persistent, ca. 1 mm long. Calyx 5-lobed, the lobes broadly lanceolate, joined briefly at the base, ca. 1.5 × 0.5–0.75 mm, sparsely pubescent with mixed simple and bifurcate hairs abaxially, reflexed at anthesis. Petals 5, unguiculate, the claw filiform, ca. 4–6 mm long, glabrous, the lamina broadly ovate, ca. 1–1.5 × ca. 1 mm, the lower lamina margin divided into 2 rounded lobes, marginal cilia not present, the upper lamina margin retuse to emarginate, the adaxial surface with 2 obscure fleshy teeth, the abaxial surface without an appendage. Androgynophore to ca. 3 mm tall, glabrous; staminal column campanulate, ca. 1 mm tall; stamens 5, anthers 3-thecate, filaments ca. 0.25 mm long; staminodia 5, lobes ± triangular, ca. 0.33 × 0.33 mm, fleshy. Ovary globose, style simple, short. Capsules septicidally dehiscent, ovoid, ca. 7 × 5 mm, spiny, capsule surface minutely pubescent with simple multicellular hairs, capsule spines sparingly and minutely pubescent with simple antrorse hairs and terminating in a hyaline hair. Seeds narrowly ellipsoid, ca. 4 × 2 mm, glabrous.

#### Etymology.

The specific epithet honors Nathaly Guerrero, who has traveled the world but remains firmly attached to Colombia, her native country.

#### Distribution and habitat.

At present, known only from the banks of quebradas (ravines) in Mpio. Puerto Berrío, Antioquia, Colombia; 150–300 m alt. The type locality is 10–12 km NW of Puerto Berrío on the Río Magdalena and ca. 5 km due west of the Río San Bartolomé.

#### Additional material examined.

Colombia. • **Antioquia**: Municipio Puerto Berrío, vía Puerto Berrío-Yondó, 35 km NE de Puerto Berrío, sitio Bodegas, quebrada San Juan de Bedout, 150 m alt., 6°32'N, 74°38'W, 18 Oct 2001 (fl, fr), *R. Callejas & F.A. Cardona 12549* (MO [MO-3095808 as image!]).

#### Discussion.

*Ayenia nathaliae* like *A. saligna* is riparian and both species have narrow leaf blades, which otherwise is atypical for *Ayenia* s.str., a clade restricted to the Americas (Sharber et al. in press). This leaf type may represent an adaptation to their shared habitat. Most species of *Ayenia* s.str. are restricted to xerophytic or dry habitats and have much broader leaf blades. While both these riparian species are endemic to the Magdalena Valley of Antioquia and are found at more or less the same elevation, they occur in different river basins. The latter described from the mouth of the Río Mata where it enters the Río Porce, has also been found (*R.F. Gómez et al. 5923*, F [V0318055F as image!]) at the mouth of the Río Toná where it enters the Río Anori (see Restrepo-Santamaria et al. 2023 for a description of this latter region, the upper sub-basin of the Río Nechí, and on-going threats to its biodiversity) and is currently known from the Bajo Cauca and Nordeste subregions of Antioquia. *Ayenia nathaliae* is found on the banks of quebradas (ravines) that feed into the Río Magdalena in the Magdalena Medio subregion of Antioquia.

Morphologically, *Ayenia nathaliae* is clearly allied with *A. saligna*, *A. stipularis* Triana & Planch., *A. cuatrecasasii* Cristóbal (published as “*cuatrecasae*”), and *A. mastatelensis* Cristóbal & N. Zamora, the last four species assigned to *A.* subsect. *Stipulares* (Cristóbal 1960, 1962; Cristóbal et al. 1992; Dorr 1996). All five species share a shrubby to arborescent habit and foliar nectaries. Characters related to inflorescences and flowers, including those originally used to define the subsection (i.e., terminal inflorescences and flowers lacking a dorsal appendage), however, are not shared by all these species and cannot be used now to define this subsection. Although *A. stipularis* (type of the subsection) only has terminal inflorescences, *A. cuatrecasasii*, *A. nathaliae*, and *A. saligna* have axillary flowers or inflorescences, and *A. mastatelensis* has both axillary and terminal inflorescences. Similarly, while the petals of *A. stipularis* and *A. nathaliae* lack a dorsal appendage (terminology following Dorr 1996), the petals of the other three species have dorsal appendages.

Given that only habit and foliar nectaries now appear to define *Ayenia* subsect. *Stipulares*, its boundaries and relationships with two other subsections of the genus are unclear and probably cannot be confidently resolved without additional molecular sampling. *Ayenia* subsect. *Praeclarae* Cristóbal also includes only arborescent species with foliar nectaries. Although this latter subsection was monotypic when first described (Cristóbal 1960), a second species *A. klugii* Cristóbal & Arbo subsequently was included (Cristóbal and Arbo 1971) and only petal blades notched at the apex seem to be morphological characters that distinguish this subsection from subsection *Stipulares*. Similarly, the arborescent habit and foliar nectaries of *A. conciliata* Cristóbal, a Mexican species found in cloud forest, suggest that it could be related to both subsections *Praeclarae* and *Stipulares* (Cristóbal 1969; Cristóbal and Arbo 1971; Cristóbal et al. 1992; Dorr 1996). Cristóbal (1969) created the monotypic *A.* subsect. *Conciliatae* Cristóbal to accommodate *A. conciliata*. However, the unlobed rhomboidal petal lamina of *A. conciliata*, which more closely resembles in shape the petal lamina characteristic of *A.* sect. *Ayenia*, is fundamentally different from the petal lamina of the species assigned to subsections *Praeclarae* and *Stipulares*, which were clearly misplaced in *A.* sect. *Ayenia* by Cristóbal (1960) as they are morphologically allied to *A.* sects. *Cybiostigma* (Turcz.) Griseb. and *Leiayenia* Griseb. This division based on petal morphology is supported by the molecular phylogeny presented by Sharber et al. (in press). Thus, petal morphology suggests that *A. conciliata* was correctly placed in *A.* sect. *Ayenia* by Cristóbal (1969) and probably is not closely related to subsections *Praeclarae* and *Stipulares*. If this is the case, then there may have been an independent origin of the foliar nectary in *A. conciliata*.

Although molecular sampling is incomplete (*Ayenia conciliata*, *A. cuatrecasasii*, *A. klugii*, and *A. nathaliae* have not been sequenced), Sharber et al. (in press) determined that *A.* subsect. *Praeclarae* Cristóbal was weakly supported as sister to all the other species of *Ayenia* s.str. that they sampled. They further presented molecular evidence that subsections *Praeclarae* and *Stipulares* represent early-diverging sister lineages within the genus occupying mesic habitats in northern South America and Central America that appear more similar to habitats known for species of *Byttneria* than for most species of *Ayenia* s.str. The implication, as articulated by Sharber et al. (in the press) is that the *Ayenia* clade originated in wet tropical forests of South America and later diversified into drier habitats and higher latitudes.

Although *Ayenia nathaliae* is tentatively assigned here to *A.* subsect. *Stipulares*, this subsection may eventually be united with *A.* subsect *Praeclarae* (the two subsections have equal nomenclatural priority). Morphology suggests the two subsections are the same and the molecular evidence cited above for the sister relationship of subsect. *Praeclarae* is not strong. Floral morphology suggests *A.* subsect. *Conciliatae* probably represents a different lineage. Again, additional molecular evidence would be informative.

##### Key to the Colombian species of *Ayenia* with foliar nectaries

**Table:** 

1	Leaf blades lanceolate or narrowly oblong, narrow, < 1.0 cm wide	**2**
–	Leaf blades elliptical, ovate-lanceolate or obovate, broad, > 1.0 cm wide	**3**
2	Leaf blades lanceolate, 2.5–7.5 × 0.5–1.0 cm, margin denticulate its entire length, apices long acuminate; bases of petal lamina lobes ciliate and apices with a terete appendage	***A. saligna***
–	Leaf blades narrowly oblong 0.9–3.3 × 0.2–0.4 cm, margin entire or occasionally toothed toward the apex, apices abruptly apiculate; bases of petal lamina lobes lacking cilia and apices without an appendage	***A. nathaliae***
3	Inflorescences terminal, many-flowered; leaf blades glabrous	***A. stipularis***
–	Inflorescences axillary, few-flowered; leaf blades minutely and sparingly pubescent	**4**
4	Margin of leaf blades slightly serrate its entire length, apices acuminate; bases of petal lamina lobes lacking cilia	***A. cuatrecasasii***
–	Margin of leaf blades with irregularly-spaced teeth confined to the apical third, apices long acuminate; bases of petal lamina lobes ciliate	***A. klugii***

##### New combinations in *Ayenia*

Several species of *Byttneria* described from Colombia were overlooked in the catalogs published by Idárraga Piedrahíta et al. (2011) and Bernal et al. (2016) for the floras of Antioquia and Colombia, respectively. These names, and at least one other, were not transferred to *Ayenia* by Christenhusz et al. (2018) and the combinations are made here. This raises the number of species of *Ayenia* known to occur in Colombia to 28 of which five have foliar nectaries.

### Ayenia idroboi

Taxon classification

Plantae

MalvalesMalvaceae

(Cristóbal) Dorr comb. nov.

118979CC-7C8A-5F84-987C-89D7A58A296C

urn:lsid:ipni.org:names:77378629-1

Byttneria idroboi Cristóbal, Bonplandia (Corrientes) 4: 383, fig. 97. 1976 (“*Idroboi*”). Type: Colombia. • Caquetá [“Comisaría del Caquetá”]: Florencia, Quebrada del Río Hacha, 400–450 m alt., 1 Apr 1940 (fl), *J. Cuatrecasas 8962* (holotype: COL [COL000002538 as image!]; isotype: US [00102135]!).

#### Distribution.

Endemic to Colombia, where it is known only from Caquetá.

### Ayenia macrophylla

Taxon classification

Plantae

MalvalesMalvaceae

(Kunth) Dorr comb. nov.

FF97EBE3-1CEF-5778-8D20-0DB4C4B0C0A7

urn:lsid:ipni.org:names:77378630-1

Byttneria macrophylla Kunth, in H.B.K., Nov. Gen. Sp. [qu] 5: 315. 1823 [“1821”] (“*Büttneria*”); Ibid. [fol] 5: 245. 1823 [“1821”] (“*Büttneria*”). Type: Colombia. • Tolima: Honda, [ca. 250 m alt.], s.d. (fl), *F.W.H.A. Humboldt & A.J.A. Bonpland* [*1684*] (lectotype as “holotipo”, designated by Cristóbal (1976: 118): P-HBK [IDC microfiche 133/4!]; isolectotypes: G, P [P02286199 as image!]).
Byttneria mollis Kunth, in H.B.K., Nov. Gen. Sp. [qu] 5: 314, tabs. 481a, 481b. 1823 [“1821”] (“*Büttneria*”); Ibid. [fol] 5: 244, tabs. 481a, 481b. 1823 [“1821”] (“*Büttneria*”), non Ayenia mollis Brandegee, Univ. Calif. Publ. Bot. 3: 385. 1909. Type: Colombia. • “prope Santa Fe de Bogota”, ca. 2660 m alt., s.d. (fl), *J.C.B. Mutis s.n*. (lectotype as “holotipo”, designated by Cristóbal (1976: 120): P [P02286203 as image!]; isolectotype: P-HBK [IDC microfiche 133/3!]).
Ayenia aculeata Mutis ex Kunth, in H.B.K., Nov. Gen. Sp. [qu] 5: 314. 1823 [“1821”]; Ibid. [fol] 5: 244. 1823 [“1821”], nom. nud. pro syn.
Byttneria arguta Triana & Planch., Ann. Sci. Nat., Bot., sér. 4, 17: 331. 1862 (“*Büttneria*”). Type: Colombia. • Tolima: “Coyaima, vallée du Magdalena”, s.d. (immature fr), *J. Goudot s.n*. (lectotype as “holotipo”, designated by Cristóbal (1976: 120): P [P06723209 as image!]).

#### Distribution.

Found in Colombia, Venezuela, and Peru.

### Ayenia morifolia

Taxon classification

Plantae

MalvalesMalvaceae

(Triana & Planch.) Dorr comb. nov.

67A8C6A5-0596-5994-A9CC-B951C2C14ED9

urn:lsid:ipni.org:names:77378631-1

Byttneria morifolia Triana & Planch., Ann. Sci. Nat., Bot., sér. 4, 17: 330. 1862 (“*Büttneria*”). Type: Colombia. • Meta: “Llano de San Martín, bassin du Meta”, s.d. (fl, immature fr), *J. Goudot s.n*. (lectotype as “holotipo”, designated by Cristóbal (1976: 79): P [P02286205 (= F neg. no. 35438) as image!]; isotype: GOET [GOET011187 as image!]).

#### Distribution.

Endemic to Colombia, where it is known only from Meta.

## Supplementary Material

XML Treatment for Ayenia nathaliae

XML Treatment for Ayenia idroboi

XML Treatment for Ayenia macrophylla

XML Treatment for Ayenia morifolia

## Additional information

### Artificial Intelligence (AI) use

The author accepts full responsibility for the content of the manuscript, including the disclosure of any use of AI.

No AI tools were used in the preparation of this manuscript.

### Author ORCIDs

L.J. Dorr https://orcid.org/0000-0001-7157-363X

### Data availability

All of the data that support the findings of this study are available in the main text.
